# Typhoid Fever in Chile 1969–2012: Analysis of an Epidemic and Its Control

**DOI:** 10.4269/ajtmh.18-0125

**Published:** 2018-07-25

**Authors:** Claudia Marco, Iris Delgado, Claudio Vargas, Ximena Muñoz, Zulfiqar A. Bhutta, Catterina Ferreccio

**Affiliations:** 1 Escuela de Medicina, Pontificia Universidad Católica de Chile, Santiago, Chile;; 2 Centro de Políticas Públicas, Faculty of Medicine, Universidad del Desarrollo, Santiago, Chile;; 3 Departamento de Matemática y Ciencia de la Computación USACH, Santiago, Chile;; 4 Secretaría Regional Ministerial de Salud de Arica y Parinacota, Arica, Chile;; 5 Centre for Global Child Health, The Hospital for Sick Children, Toronto, Canada;; 6 Dalla Lana School of Public Health, University of Toronto, Toronto, Canada;; 7 Center of Excellence in Women and Child Health, The Aga Khan University, Karachi, Pakistan;; 8 Advanced Center for Chronic Diseases, ACCDiS, Pontificia Universidad Católica de Chile. Santiago, Chile

## Abstract

From 1975 to 1983, a large epidemic of typhoid fever (TF) affected the metropolitan region (MR) of Chile (incidence rate [IR] of 219.6 per 10^5^ in 1983). In 1983–1984, interventions were implemented focusing on person-to-person transmission (vaccination, food handlers’ control, and mass communication) and regulations to control irrigation waters containing fecal contaminates. In 1991, a second intervention was quickly implemented to avoid the cholera epidemic affecting neighboring countries (total prohibition of growing or selling crops in the MR). We explored the potential impact of these interventions on the epidemic. We created a yearly database of the MR TF cases, population, and contextual factors of TF from 1969 to 2012. We first analyzed the epidemic (Joinpoint regression), identified predictors of TF (Poisson multiple regression), and then analyzed the effect of the interventions (interrupted time series model). The main predictor of the TF epidemic was the rate of unemployment. In relation to the 1983–1984 person-to-person interventions, TF came down by 51% (95% confidence interval [CI]: 30.2–65.0%) and continued to decrease at a rate of 10.4% (95% CI: 5.8–15.6%) per year until 1991. In 1991, with the strong environmental control of the sewage-irrigated crops, TF further decreased by 77% (95% CI: 69.0–83.1%) and continued decreasing thereafter at 13% (95% CI: 11.3–15.6%) per year until the end of the study period. Today, 40 years after the epidemic, TF is a rare disease in the MR of Chile.

## INTRODUCTION

From 1950 to 1976, typhoid fever (TF) was highly endemic in Chile and did not seem to respond to improvements in household coverage of safe drinking water or sewage collection; in 1977, a large epidemic cycle began (Supplemental Figure 1).^1^ This has been the largest TF epidemic reported in Latin America, occurring in a country with the best health indicators in the region.^2,3^ The highest incidence rates (IRs) were seen in the metropolitan region (MR), one of Chile’s 16 geographical regions, located in the center of the country. The MR includes Santiago, the country’s capital city and seat of government, home to 40% of the national population; this region had better sanitary conditions than the rest of the country.^4,5^ The TF epidemic was attributed to various causes, including person-to-person transmission of *Salmonella enterica* serovar Typhi (ST), the short cycle associated with poor personal hygiene,^4^ and deterioration of safe food handling practices,^2^ deterioration of drinking water chlorination,^6,7^ or the use of untreated sewage in irrigation systems.^8,9^ Important public health interventions directed to the control of TF were first put in place in 1983–1984.^10,11^ In 1991, the government implemented a second public health intervention to avoid the cholera epidemic affecting neighboring countries.^12,13^ Our aim was to evaluate the association of these interventions with changes in the IR of TF. We collected all available data and used sound statistical analysis, to evaluate this association, which could inform policy makers in areas of high TF incidence.

**Figure 1.**
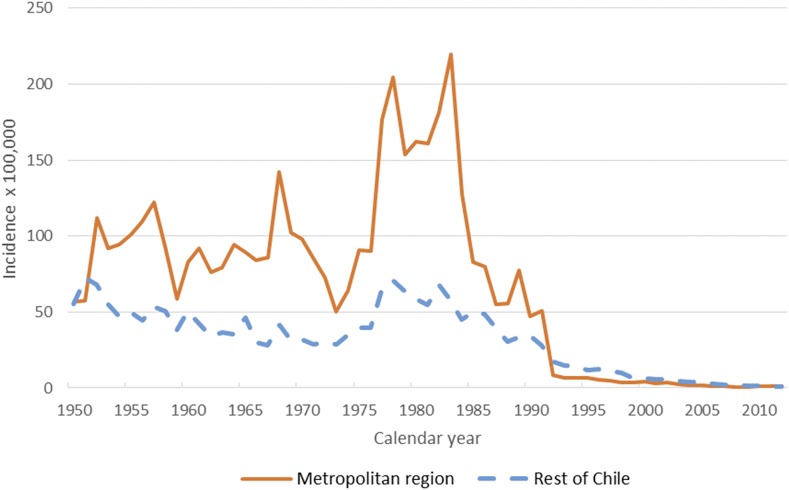
Typhoid fever incidence in the metropolitan region (MR) and the rest of Chile, 1950–2012. The orange line depicts typhoid incidence trends per 100,000 persons in the MR of Chile. The hashed blue line shows the trend in typhoid incidence per 100,000 persons in the rest of Chile.

## METHODS

We implemented an ecological study using a time series analysis of TF in the MR, comprising group-level data (TF IRs, IR), environmental data (sanitation), and contextual data (socioeconomic metrics). The unit of analysis for this study was the MR, and the unit of observation was each year between 1969 and 2012 (44 time points). We collected the age–gender distribution of the population and of the TF cases reported and TF determinants (sanitation and socioeconomic).

We used interrupted time series (ITS) analysis to model the two interventions implemented for the control of TF in the MR.^14^ The study was approved by the Pontificia Universidad Católica de Chile Ethical Committee.

### Population and typhoid fever data.

We obtained the MR population data by age and year between 1950 and 2012 from the national census and intercensal projections,^15^ and the number of new TF cases (Table 1) and deaths (Supplementary Figure 1), by age, place of residence, month and year were obtained from the compulsory transmissible diseases notification system of the Ministry of Health (MoH)^16^. We analyzed seasonality (Supplemental Figure 2), age distribution (Supplemental Figure 3), and *S. enterica* serotype distribution (Supplemental Figures 4 and 5). We analyzed the following environmental risk factors of enteric infections: drinking water, sewage, final wastewater treatment, sanitary inspections of food handling, inspections of irrigation waters, measurements of residual chlorine in drinking water, and vegetable production (Supplemental Figures 6–8).^17–21^ We also included the following socioeconomic markers: poverty, schooling, gross domestic product, Human Development Index, public health expenditure, and unemployment (Supplemental Figures 9–11).^22–25^

**Table 1:** Metropolitan region population, TF cases, and incidence rates by year

Year	Cases	Population	IR × 10^5^	Intervention 1	Intervention 2	Unemployment %
1969	3,463	3,381,181	102.42	0	0	6.15
1970	3,408	3,478,667	97.97	0	0	7.13
1971	3,007	3,553,922	84.61	0	0	5.50
1972	2,640	3,629,177	72.74	0	0	3.78
1973	1,865	3,704,432	50.35	0	0	4.63
1974	2,424	3,779,687	64.13	0	0	9.65
1975	3,500	3,854,943	90.79	0	0	16.18
1976	3,545	3,927,154	90.27	0	0	16.78
1977	7,070	3,999,366	176.78	0	0	13.23
1978	8,334	4,071,578	204.69	0	0	14.00
1979	6,358	4,143,789	153.43	0	0	13.55
1980	6,827	4,216,001	161.93	0	0	11.75
1981	6,936	4,301,778	161.24	0	0	11.08
1982	7,954	4,387,555	181.29	0	0	22.10
1983	9,825	4,473,332	219.63	0	0	22.23
1984	5,819	4,559,109	127.63	1	0	19.23
1985	3,848	4,644,886	82.84	1	0	16.35
1986	3,780	4,749,844	79.58	1	0	13.50
1987	2,675	4,854,803	55.10	1	0	12.25
1988	2,774	4,959,761	55.93	1	0	10.98
1989	3,938	5,064,719	77.75	1	0	9.13
1990	2,428	5,169,678	46.97	1	0	9.55
1991	2,690	5,271,098	51.03	1	0	7.43
1992	447	5,372,519	8.32	1	1	6.00
1993	364	5,473,940	6.65	1	1	6.28
1994	361	5,575,360	6.47	1	1	6.78
1995	389	5,676,781	6.85	1	1	6.63
1996	302	5,769,478	5.23	1	1	6.23
1997	273	5,862,175	4.66	1	1	6.63
1998	231	5,954,872	3.88	1	1	9.60
1999	207	6,047,569	3.42	1	1	9.70
2000	259	6,140,266	4.22	1	1	9.80
2001	201	6,212,770	3.24	1	1	NA
2002	212	6,285,273	3.37	1	1	NA
2003	146	6,356,534	2.30	1	1	9.30
2004	133	6,425,332	2.07	1	1	NA
2005	100	6,494,536	1.54	1	1	NA
2006	80	6,565,792	1.22	1	1	7.00
2007	63	6,640,697	0.95	1	1	NA
2008	54	6,720,663	0.80	1	1	NA
2009	35	6,804,444	0.51	1	1	10.10
2010	70	6,887,859	1.02	1	1	NA
2011	82	6,971,899	1.18	1	1	NA
2012	65	7,057,491	0.92	1	1	NA

IR = incidence rate; NA = data not available; TF = typhoid fever. Metropolitan region of Chile, 1969–2012. The presence of the intervention is marked as 1 and its absence as 0. Unemployment was included as the variable which better explained the raise of TF in the 1970s.

### Interventions to control TF.

We collected data on two interventions: the first was implemented over 2 years and was directed specifically toward controlling TF,^10,11^ the second was implemented over the course of a few months with the objective of protecting Chile from the cholera epidemic affecting neighboring countries.^12,13^

### Intervention No. 1 1983–1984.

At the beginning of 1980, the MoH, in collaboration with the World Health Organization and the University of Maryland, designed the TF Control Program; its transference to public health occurred mainly during 1983 and 1984. This TF Control Program included a series of epidemiological studies to clarify the main transmission mechanisms of TF and included an evaluation of a new oral vaccine, educating food handlers, new methods for detection of chronic carriers, and a clinical trial of a new drug for chronic carriage treatment. They found ST cultivable in 10% of the irrigation water samples studied.^8−10,26^ This result prompted the prohibition of cultivating or marketing vegetables generally consumed raw, that grew at ground level (lettuce, coriander, parsley, and radishes among others), and initiation of mass communication and education campaigns about hygienic handling of crops. The clinical trials of a new oral typhoid vaccine, with an estimated efficacy of 70%, immunized 486,319 schoolchildren aged 5–19 years, covering a large proportion (60%) of the target population, with at least one dose of the oral vaccine.^10^ The food handler management included sanitary control and education in restaurants and school kitchens; in addition, small studies of chronic carrier detection with Vi serology and treatment with ciprofloxacin were carried out.^10^

### Intervention No. 2 1991.

In early 1991, the government implemented emergency measures to avoid the propagation of the cholera epidemic affecting neighboring countries into Chile. It instituted an emergency action commission to implement and enforce control measures to prevent transmission of enteric infections in the MR and the rest of Chile. All measures were directed toward immediately stopping the long transmission cycle of TF from crop irrigation with sewage-contaminated water to crop consumption by the population. Through the joint action of the ministries of health, education, agriculture, and internal affairs, this commission intervened in improvement of water quality and irrigation practices, and further regulated the commercialization of crops. Specific measures included increasing the number of prohibited crops; banning restaurants from serving raw vegetables, raw fish, and seafood; construction of new irrigation channels separated from sewage discharges; chlorination of water channels; prohibition of any human activities near water sources with sewage discharges; and establishment of sanitary barriers in those areas.^12,13^

### Statistical analysis.

The first step was to identify the main risk factors of the TF epidemic as a means to identify and control possible confounders of the interventions. We evaluated the association of individual risk factors with the annual IR of TF by bivariate correlations, using r-Pearson correlation (Supplemental Table 2). Significant factors were included in a Poisson multiple regression model. We then conducted a Joinpoint regression of TF over the study period with complete TF data (1950–2012) to identify significant breaks in the trends of the incidence of the disease.^27^ The second step was to analyze the effect of the interventions using ITS analysis.^14^ Records of the interventions performed in Santiago between 1969 and 2015, to control enteric transmission, demonstrated that there were two distinct governmental interventions, the first mass intervention extended over a 2-year period—1983 to 1984—and the second was a more focused and intense intervention in the summer of 1991. Based on the timing of these two , we divided the study period into four segments: 1969–1973 or 1950–1973 (pre-epidemic), 1974–1983 (epidemic), 1984–1991 (post-intervention 1), and 1992–2012 (post-intervention 2). We used the annual IR over the study period to estimate the change in disease level (intercept terms) and in disease trend (slope terms) for each intervention period. Variables significant in the Poisson multiple regression were entered in the ITS analysis as possible confounders. For the ITS analysis, our first approach was modeling TF incidence data with a Poisson generalized linear model.^28^ Nevertheless, with Cameron–Trivedi test, we found data overdispersion^29^; therefore, we used the negative binomial regression^30^ presented here:

$$\text{log}\left(E\left[Y_{t}\right]\right)=\beta_{0}+\beta_{1}T+\beta_{2}X_{0}+\beta_{3}\left(T-T_{1}\right)X_{0}+\beta_{4}X_{1}+\beta_{5}\left(T-T_{2}\right)X_{1}+\beta_{6}X_{2}+\beta_{7}\left(T-T_{3}\right)X_{2}+\text{log}\left(\mathrm{po}p_{t}\right);$$

*Y*_*t*_: represents the number of cases of TF in year *t*,
*T*: represents the calendar times beginning with 1969 = 1; T1 is the time of the initiation of the epidemic, T2 and T3 represent the initiation of each intervention. These time points were selected based on the date of the interventions initiation,
*X*: represents the presence of the epidemic (*X*_0_) and of each intervention (*X*_1_ and *X*_2_), and
Pop_*t*_: Represents the population of Santiago in the year *t.*

We ran the full model, including the previously selected confounders and evaluated the results of each β parameter using the *z*-test. We reevaluated the model dropping variables for which β were statistically nonsignificant. The resulting models were compared using the Akaike information criteria (AIC) to select the best model. We analyzed the residuals of the selected model to detect autocorrelation, R’s autocorrelation, and partial autocorrelation functions. We estimated confidence intervals (CIs) for the change in the slope and the slope in the segment with the open statistical package R (application multcomp and function confint). We used the delta method to estimate the CIs for the TF IR per 10^5^. Statistical analyses were performed with SPSS version 23.0 (IBM Corp., Chicago, IL) and R Core Team (2017).^31^ We compared the ITS analysis for two time intervals 1969–1973, when we had complete information on contextual factors, and 1950–1973, when we only had TF data. Finally, we analyzed the decrease of TF IR in four age groups (younger than 5, 5–19, 20–34, and 35 years and older) and three study periods (1969, 1983, and 2005–2012), using a Poisson regression analysis, we included interaction terms for age and periods. Based on this model, we estimated the reduction of TF IR for each age group from the epidemic to the end of the study periods (1983 versus 2005–2012) and from the endemic to the end of the study periods (1969 versus 2005–2012).

## RESULTS

Typhoid fever in the MR was highly endemic, without a clear trend in incidence, from 1950 to 1976; in 1977, TF increased abruptly reaching its worst epidemic ever reported, which lasted until 1985. Typhoid fever incidence had a strong downward trend until 1991 and in 1992, its decline accelerated reaching, by the end of the study period, the lowest TF IR reported in the MR (Figure 1). Typhoid fever mortality rate had a clear downward trend from the mid-60s, moderately increasing during the epidemic peak, and then decreasing again in the late 1970s; although cases were still very high, case-fatality rate decreased during the epidemic years (Supplemental Figure 1). Annual cases of TF in the MR in 1969 were 3,463, in 1983 peaked to 9,825, then in 1984 fell to 5,819, in 1992 the cases fell further to 447, and by 2012 there were only 65 TF cases reported (Table 1). The marked seasonality during the high endemic and epidemic years, when TF peaked in the warm seasons, was less evident by the end of the study period (Supplemental Figure 2). The IR of TF dropped in all ages, but the size of the reduction varied significantly among the age groups; by the end of the study, the TF IR, compared with the pre-epidemic level, dropped 25 times (95% CI: 19–34) among 0–4 year olds, 116 times (95% CI: 100–134) among 5–19 year olds, 121 times (95% CI: 102–143) among 20–34 year olds, and 51 times (95% CI: 41–63) among 35 year olds and older (Supplemental Table 1).

The available data on *S. enterica* serotypes suggest that during the epidemic, ST was predominant until 1993 (nearly 80% of cases) when it began to decrease, reaching 40% at the end of the study period. At this time, the proportion of *Salmonella* Paratyphi B increased reaching up to 60% of cases, whereas *Salmonella* Paratyphi A ran from 0% to 2% throughout the period (Supplemental Figures 4 and 5). Environmental sanitization variables had not deteriorated before nor during the epidemic. Coverage of drinking water and sewage collection were improving during the epidemic; final sewage treatment was not implemented until 2002 in the MR, when the disease had been controlled (Supplemental Figures 6–9). Contrarily, most socioeconomic indicators deteriorated during the epidemic period (Supplemental Figures 9–11); in the Poisson multiple regression, only unemployment was selected (*R*^2^ = 0.967; B = 3.56 95% CI: 1.19–5.93, *P* = 0.001).

**Figure 2.**
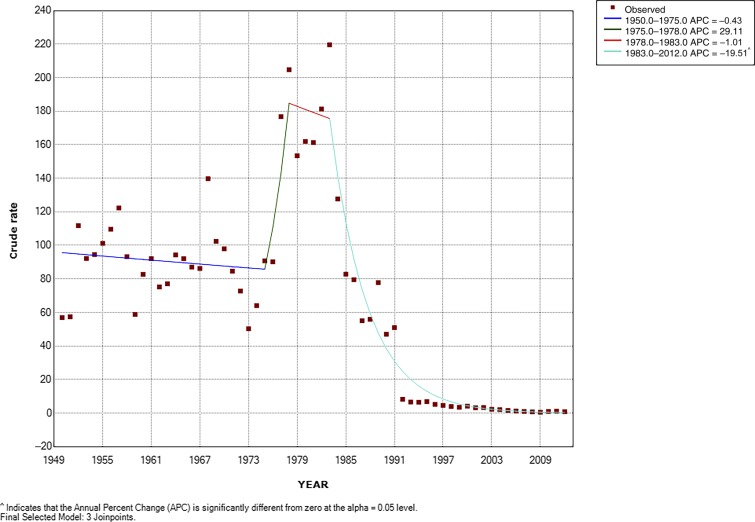
Typhoid fever (TF) incidence trend in the metropolitan region of Chile Joinpoint regression 1950–2012. Best joinpoint model. ^ indicates that the average percent change (APC) is significantly different from zero at alpha = 0.05 level. The squares represent the observed rates per 100,000 persons of TF across the study period (1950–2012). The dark blue line is the joinpoint model from 1950 to 1975. The dark green line is the joinpoint model from 1975 to 1978. The red line shows the joinpoint model from 1978 to 1983. The turquoise line shows the joinpoint model from 1983 to 2012. Note: The joinpoint model is a Poisson model that, unlike the interrupted time series, does not incorporate level changes and only changes in the slope. It looks for points where the slope breaks. To estimate a slope, it requires at least two intermediate points between breakpoints.

Without including external a priori information, the Joinpoint regression analysis from 1950 to 2012 showed four statistically significant changes in trend: a stable period from 1950 to 1975, a rapid increase from 1975 to 1978, a short stable period from 1978 to 1983, and then a rapid and significant drop from 1983 to 2012 (Figure 2).

**Figure 3.**
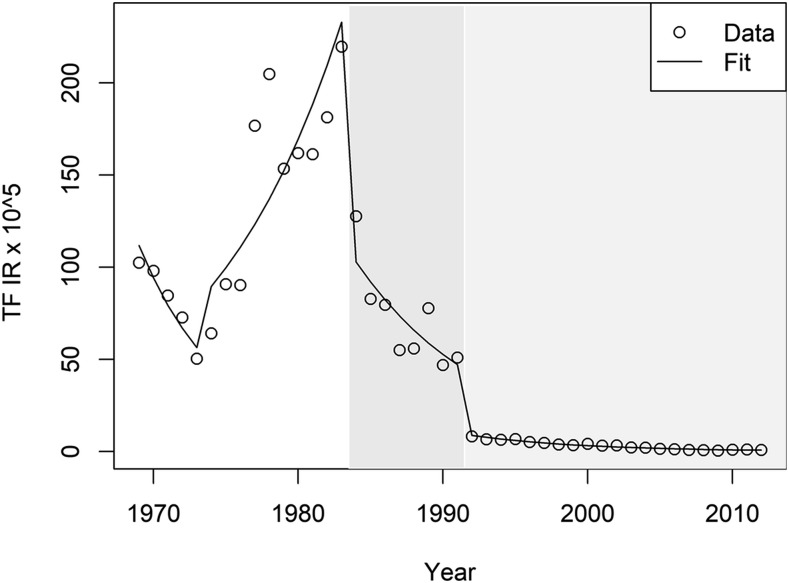
Typhoid fever (TF) observed incidence rates (IRs) in the metropolitan region and interrupted time series model fit. Typhoid fever observed IRs per 100,000 persons between 1969 and 2010 are shown with the open circles. Predicted TF IRs within the same time period per 100, 000 persons is shown as a line of best fit. Gray bands represent the area of effect of each intervention. The darkest band represents Intervention 1.

The TF IR pre- and post-intervention periods are presented in Table 1. We also present the trend of unemployment in the study period, which was the only contextual variable significantly associated with TF in the multivariable model (Table 1). The best negative binomial regression model, according to the AIC, included TF and the two interventions; other variables were not significant. The results of the model for the four study segments are presented in Table 2 and Figure 3. The pre-epidemic period is the starting point of the series; TF IR was higher at the beginning of this segment than at the beginning of the next, with a negative trend in the entire pre-epidemic period. The epidemic period begins with TF rates of 56.4 cases per 100,000 persons reaching 232.8 per 100,000 at the onset of the next period with an important increase in its level and trend. Post-intervention no. 1, there was a 51% reduction in the level (calculated from second column of Table 2) and more importantly, a change in the trend (calculated from second column of Table 2). In the whole segment, the IR was decreasing at a pace of 10.4% per year (calculated from second column of Table 2). Post-intervention no. 2, the drop in the level was even higher (−1.48; 78% reduction), with a lower but nonsignificant drop in the slope; nevertheless, in the whole segment, the IR decreased to 13% per year (Table 2). This model had a good fit and the analysis of residuals showed a random distribution (Figure 3). When we ran the ITS model with the whole TF data from 1950 to 2012, the model parameters did not change, with very similar predicted values for the relevant study period (Supplemental Table 2).

**Table 2:** Changes in the TF incidence rate level and trend and association with the interventions

Study segments	Point change in level	Level at segment onset TF incidence rate per 10^5^	Change in slope in the segment	Slope in segment
Pre-epidemic	−6.62*	132.6	*	−0.17
[95% CI]	[−6.99, −6.23]	[66.29, 198.91]	–	[−0.29, 0.06]
Epidemic	0.36	56.42	0.28	0.11
[95% CI]	[−0.03, 0.73]	[35.82, 77.02]	[0.15, 0.4]	[0.06, 0.15]
Intervention 1	−0.71	232.82	−0.22	−0.11
[95% CI]	[−1.05, −0.36]	[167.63, 298.01]	[−0.28, −0.15]	[−0.17, −0.06]
Intervention 2	−1.48	47.13	−0.03	−0.14
[95% CI]	[−1.78, −1.17]	[32.52, 61.74]	[−0.09, 0.03]	[−0.17, −0.12]

CI = confidence interval; TF = typhoid fever. Metropolitan region of Chile, 1969–2012.

* Initial intercept.

## DISCUSSION

Our aim was to analyze the trend of TF in the MR from 1969 to 2012 and to evaluate its association with the public health interventions of 1983–1984 and 1991. We collected a large body of data to understand the trend of TF, including the causes of the 1977–1984 epidemic. We concluded that the main explanatory factors were the long cycle of transmission of TF through the contamination of irrigation waters by sewage, worsened by the intensification of person-to-person transmission during an economic crisis, with unemployment being its single strongest marker.

In Santiago, sewage collection coverage was increasing rapidly before, during, and after the epidemic years. Nonetheless, all sewage collected from the households was discharged untreated into the water courses used for irrigation of the city agricultural lands, thus increasing the enteric contamination of the crops eaten by the citizens. Because of this return of the contamination through the crops, the sewage collection did not benefit the Santiago population. Between 1969 and 1976, the coverage of sewage collection from homes increased by 75% in Chile. By 1982, the MR reached 82% and Chile 70% of sewage coverage. Since sewage sanitation in Santiago was implemented in 2010, the increase in sewage collection from 1969 to 1976 augmented the microbiologic burden in the irrigation waters of Santiago by at least 75% immediately before the epidemic years. This recirculation of sewage had been alerted a century before: “The present water closet system, with all its boasted advantages, is the worst that can generally be adopted, briefly because it is a highly extravagant method of converting a mole-hill into a mountain. It merely removes the bulk of our excreta from our houses to choke our rivers with foul deposits and rot at our neighbors’ door. It introduces into our houses a most deadly enemy…”—chemist quoted in the Scientific American, 1869.^32^

The TF epidemic coincided with increases of other enteric infections, in particular hepatitis A,^6,9,33−36^ suggesting a broad enteric contamination in the MR of Santiago.^1,2,4,6,9^ Unemployment is a complex variable representing a summary of the underlying socioeconomic and political crisis; it was clearly associated with the initiation of the epidemic as other authors also noted.^7^ The abrupt increase in the unemployment in Santiago (reaching 19% of the population), a marker of an important socioeconomic crisis, resulted in an increase in person-to-person transmission of enteric infections, in turn rapidly increasing the force of infection in the city causing the huge epidemic.

The 1983–1984 TF Control Program was associated with the break of the TF epidemic, which returned to the previous endemic level and the 1991 cholera prevention measures were associated with the definitive control of TF which became a sporadic disease in the MR.

The 1983–1984 intervention placed a higher emphasis on interrupting person-to-person transmission of ST, by vaccination of schoolchildren and food handlers, increase of food handling controls, and chronic carrier identification. They also conducted actions to prevent the environmental contamination and the long cycle of transmission. The 1983–1984 intervention required community participation (in vaccination programs) and changes in behavior of the public and providers. Contrarily, the emphasis of the 1991 intervention was in the interruption of the environmental transmission of enteric infections by strict control of irrigation waters and crops cultivation, transport, and marketing, forcefully interrupting the contact of sewage-contaminated items with the public; its success did not depend on the public’s willingness to follow the health recommendations.^12,13,33,35^ In association with the 1983–1984 interventions, TF came down by 51% (95% CI: 30.2–65.0%) and kept decreasing by 10.4% (95% CI: 5.8–15.6%) per year until 1991. In 1991, TF further decreased by 77% (95% CI: 69.0–83.1%) and continued decreasing thereafter at 13% (95% CI: 11.3–15.6%) per year until the end of the study period. Today, 40 years after the epidemic, TF is a rare disease in the MR of Chile. This presents strong evidence that the environmental contamination with ST was the main cause of the high endemicity of TF in the MR. The socioeconomic crisis which preceded the epidemic or hyper-endemic years, as many authors called the 1977–1983 period, offered the conditions to aggravate person-to-person transmission of ST, increasing the burden of infection in the irrigation waters and crops, thus canceling out the benefits of households sanitation. The sewage contamination of irrigation waters explains the epidemiology of the disease in the MR, with high IRs in all socioeconomic sectors, independently of the availability of drinking water or connection to sewage systems.^1^

### How do we compare with previous studies?

We were able to demonstrate that the working hypothesis of some epidemiologists was correct—that the high endemicity since the early 1950s of TF in the Chilean MR of Santiago was caused by sewage contamination of irrigation waters. In 1940, Gustavo Molina, in reference to a 1939–1940 TF outbreak in the MR, concluded that the evidence that crops contaminated by sewage irrigation is one of the main mechanisms that “opens a road for sanitary action and, more importantly, places the sewage problem of Santiago at the forefront of priorities for the sanitary organization.”^37^ Borgoño, in 1958, reviewed the epidemiology of TF in the MR; he described the decreasing trend in case-fatality rate between 1940 and 1949 (19.3% and 13.9%), later markedly accelerated by the introduction of chloramphenicol from 1950 to 1956 (6.5–1.5%). Regarding causality, he pointed out that 50% of the population was connected to sewer lines whose waters were used for crop irrigation in the MR; he concluded that TF endemicity was largely attributable to environmental transmission, urging the authorities to develop an integral sanitary program to definitively control enteric infections in the region.^38^ In 2007, Laval, in a historical review of TF in Chile, concluded that “although the hypothesis of environment contamination as the cornerstone in typhoid persistence was present since the recognition of the disease in 1894, it was faced efficiently and perhaps in a definite manner only almost 100 years later.”^1^ Cabello gave most of the causal weight of the epidemic to the lack of chlorination of drinking water.^6,7^ Drinking water chlorination cannot explain the cause nor the control of the TF epidemic because the MR reached the highest national coverage of chlorinated drinking water and yet presented the highest rates TF since 1950 (Figure 1). In the MR, the safety of the provision of drinking water was not questioned because the most sensitive indicators (infant mortality)^32^ were not affected during this epidemic and ST has never been isolated from drinking water. Contrarily, a molecular study of the *Salmonella* Typhi isolated from the irrigation waters of Santiago and from clinical cases in the summer of 1983, that is, during the epidemic, demonstrated that the same molecular type of *Salmonella* Typhi was present in the water and in patients.^39^ Thus, it provided further evidence for the link between sewage contamination and TF.

Others did not believe in the role of environmental transmission. Romero published a comprehensive review of the epidemiology of TF in Chile from 1931 to 1950; he stated that most transmission occurred in cities, with occasional outbreaks in rural areas, concluding that the attribution to sewage-contaminated vegetable consumption was highly overestimated. He saw the role of carriers as the main mechanism of transmission, mentioning, as a supporting argument, the lack of effect of measures such as the prohibition of growing crops or selling oysters in certain areas. He concluded that TF would be controlled both by improving sanitation and increasing the hygienic culture of the individuals.^40^ His ideas were the more prevalent conceptions in the 1970s, when the large epidemic of TF began. In this article, we demonstrated that the hypothesis of the contaminated crops was in fact correct, and that the lack of effect of certain control measures was explained by the inefficacy of the historical control policies. Only the mass interventions of 1983–1984 and markedly in 1991, effectively interrupted transmission of enteric infections by contaminated crops.

The main strengths of the study are the large series of contextual TF data and the quality of the TF reporting system, which covers the whole MR and its central management; the availability of abundant unpublished or gray literature about the Typhoid Control Program, which was coordinated by one of the authors (C. F.); the statistical model selected, which appropriately fit the data; and Chile’s very unique situation regarding TF, a quasi-experimental design in which we could analyze the factors associated with the increase and the decline of the disease in the population.

The main limitation is the observational nature of the study, in which we could not rule out that unmeasured confounders were causing the changes in the disease. Also, the 1983–1984 intervention is not an ideal clear-cut intervention; instead it extended over a broad period and consisted of various measures of different efficacy, being impossible to individualize the contribution of each factor to the control of the disease.

Another important limitation is related to our models, which did not include the dynamics of the disease.^41^ Thus, we could not estimate how much of the changes could have been explained by exhaustion of the susceptibility in the community. The latter would have come into effect in the years immediately after a large outbreak, but by itself it cannot explain the maintenance of the low TF transmission 40 years after the peak of the epidemic. The near disappearance of TF in the MR is better explained by the definitive interruption of its main transmission route.

Intervention 1 was complex, consisting of vaccination, intensive mass communication, and increases in environmental controls. It is not possible to disentangle the effects of these various components; the vaccine effect in the community may last longer than the duration of the immunological protection reached by the vaccines. Not having a dynamic model, it was not possible to estimate the duration of the effect of the particular interventions. Thus, we assumed that the first intervention changed the dynamics of the disease from then on, maintaining its effect until the end of the study period; this may represent an overestimation of its effect.

## CONCLUSION

Our findings can be applied to many areas in which sewage is discharged into irrigation waters. Our results suggest that ST could be transmitted through this mechanism and this transmission route can be effectively interrupted by short-term public health measures which do not require very large investments; these should be maintained and enforced until a structural solution, such as sewage treatment plants, is in place. We showed that the definitive control of ST transmission was achieved immediately after 1991, in concordance with a powerful government decision to completely stop the irrigation with sewage-contaminated waters.

## Supplementary Material

Supplemental figures and tables
