# Supplementary material for: Typhoid Fever in Chile 1969–2012: Analysis of an Epidemic and Its Control
Source: Am J Trop Med Hyg. 2018 Jul 25;99(3 Suppl):26–33. doi: 10.4269/ajtmh.18-0125 (PMC6128366; doi:10.4269/ajtmh.18-0125)
Supplement: Supplementary file 1 [file tpmd180125.SD1.pdf]

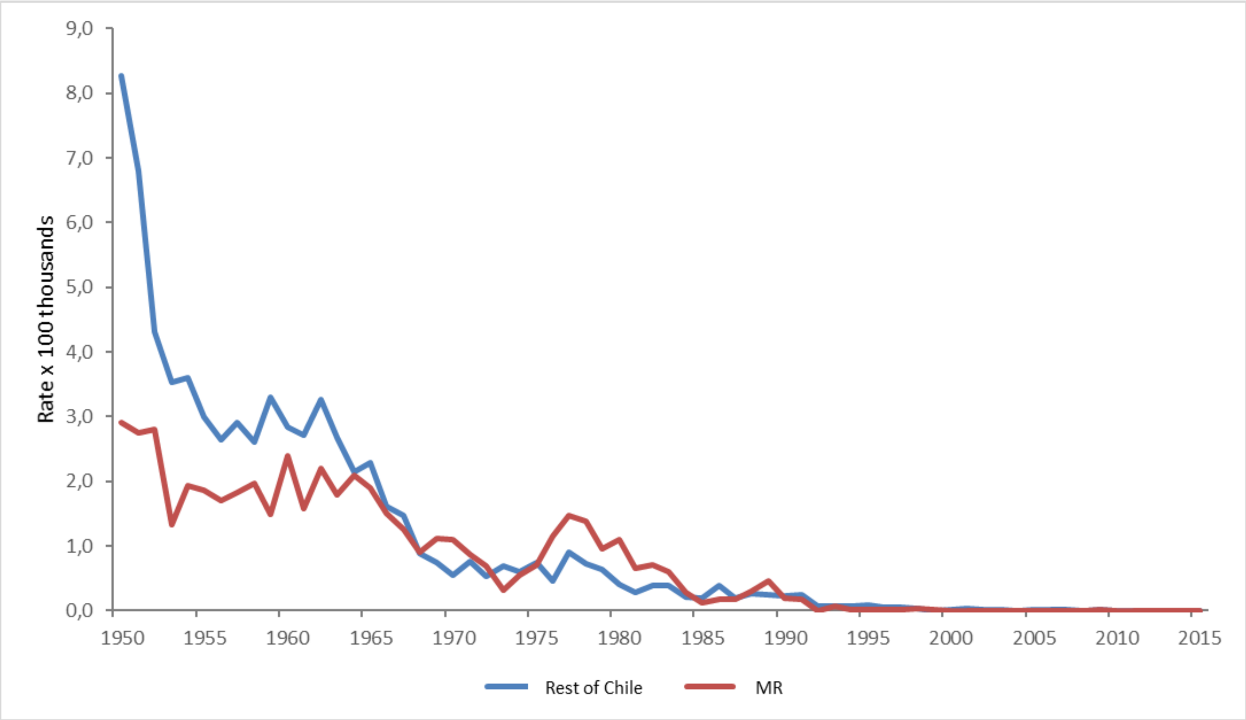

Supplementary Figure 1 Typhoid fever mortality in the Metropolitan Region and rest of Chile, 1950-2012.

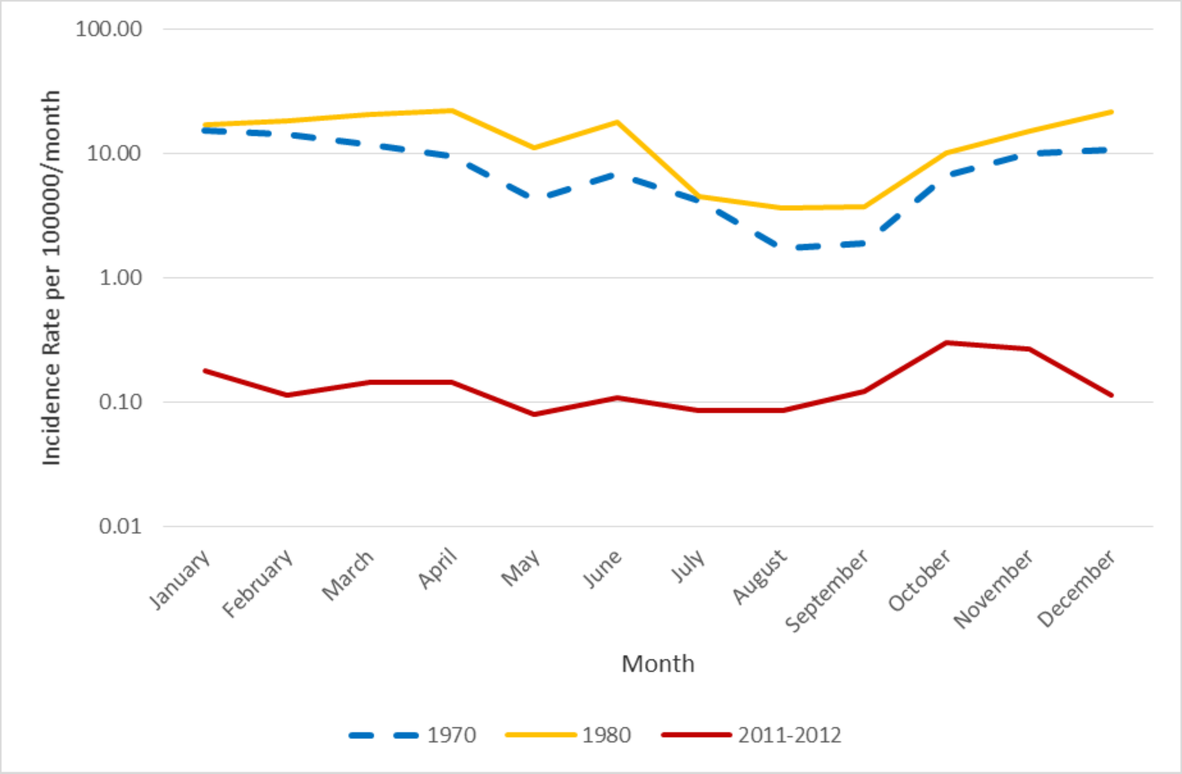

Supplementary Figure 2 Seasonality of Typhoid Fever before, during and after the epidemic. (1970-1977-2011) Monthly Incidence rates.

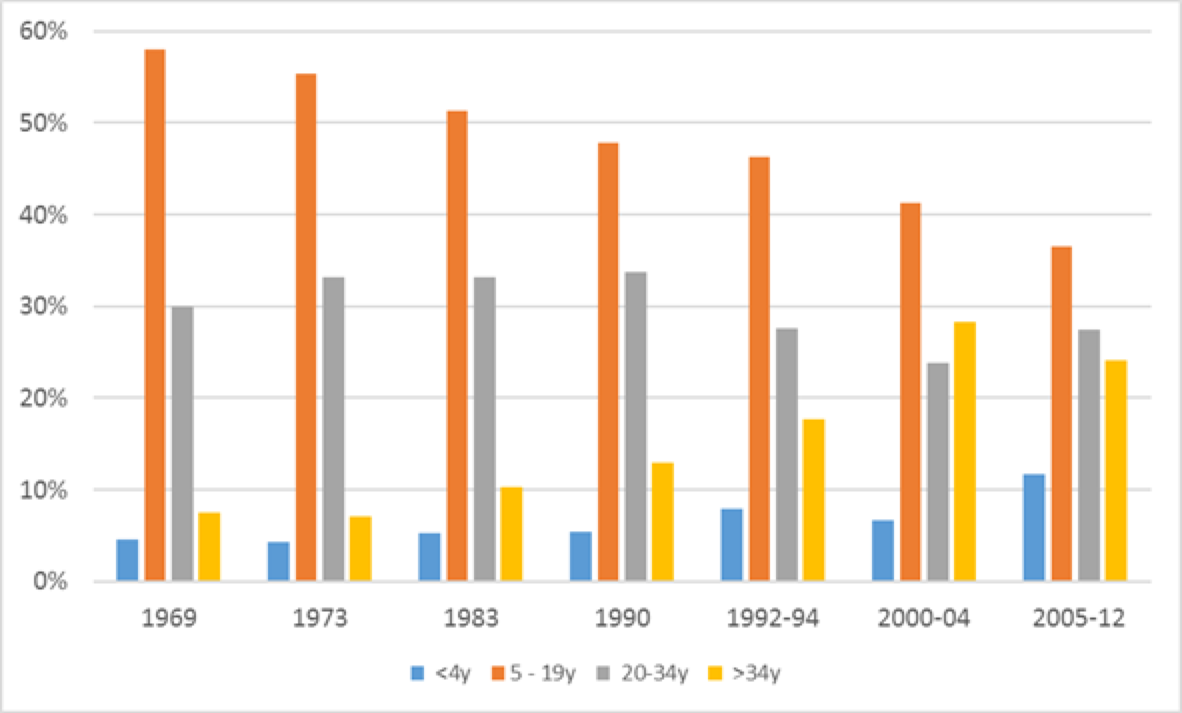

Supplementary Figure 3 Age distribution of TF cases in the MR.

Supplementary Table 1

Drop in the Incidence Rate of Typhoid Fever between Study Periods by Age Groups 1969 To 2012

Age group (years)	1983 to 2005-2012		1969 to 2005-2012	
	TF IR Reduction*	95% Conf Interv*	TF IR Reduction**	95% Conf Interv**
0-4	66.5	51.3-86.3	25.1	18.8-33.6
5-19	241.1	209.4-277.6	115.8	100.2-133.9
20-34	238.0	202.2-280.2	120.8	101.8-143.3
35 and over	129.4	107.9-154.9	50.9	41.3-62.8

*: times of reduction in the TF IR at the end of the study period with respect to the epidemic period, ** times of reduction in the TF IR at the end of the study period with respect to the endemic period.

THE POISSON MODEL FOR AGE-PERIOD EFFECT ON TF IR

```
Poisson regression                                Number of obs     =         12
                                                  LR chi2(11)       =   57624.43
                                                  Prob > chi2       =     0.0000
Log likelihood = -48.144249                       Pseudo R2         =     0.9983
```

Casos	IRR	Std. Err.	z	P>|z|	[95% Conf.	Interval]
Edadcat						
5_19	4.567149	.3798527	18.26	0.000	3.880165	5.375764
20_34	3.535312	.3039071	14.69	0.000	2.987141	4.184079
35_80	.7005245	.0712584	-3.50	0.000	.5739021	.8550841
periodo						
2	2.64761	.2419087	10.66	0.000	2.213507	3.166848
3	.0397908	.0059067	-21.72	0.000	.029746	.0532276
Edadcat#periodo						
5_19#2	.7863443	.074829	-2.53	0.012	.6525471	.947575
5_19#3	.2170249	.036001	-9.21	0.000	.1567866	.3004072
20_34#2	.7443192	.0730695	-3.01	0.003	.6140406	.9022384
20_34#3	.2080529	.0358173	-9.12	0.000	.1484689	.2915494
35_80#2	.9587374	.1104932	-0.37	0.715	.7648915	1.20171
35_80#3	.493313	.090279	-3.86	0.000	.3446258	.7061504
_cons	.0003972	.0000318	-97.81	0.000	.0003395	.0004647
ln(POB)	1	(exposure)				

Note: _cons estimates baseline incidence rate.

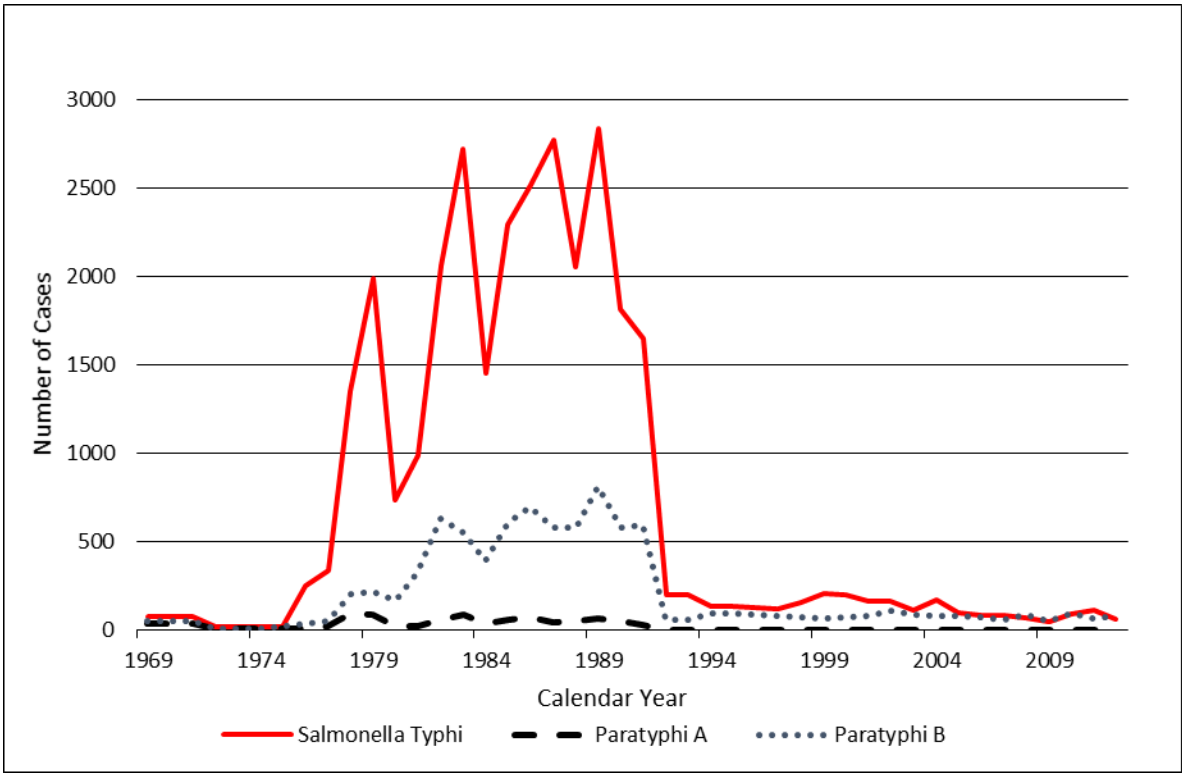

Supplementary Figure 4 Number of Salmonella enterica serovar Tyhpi, Paratyphi A and Paratyphi B isolated in Chile 1969-2012.

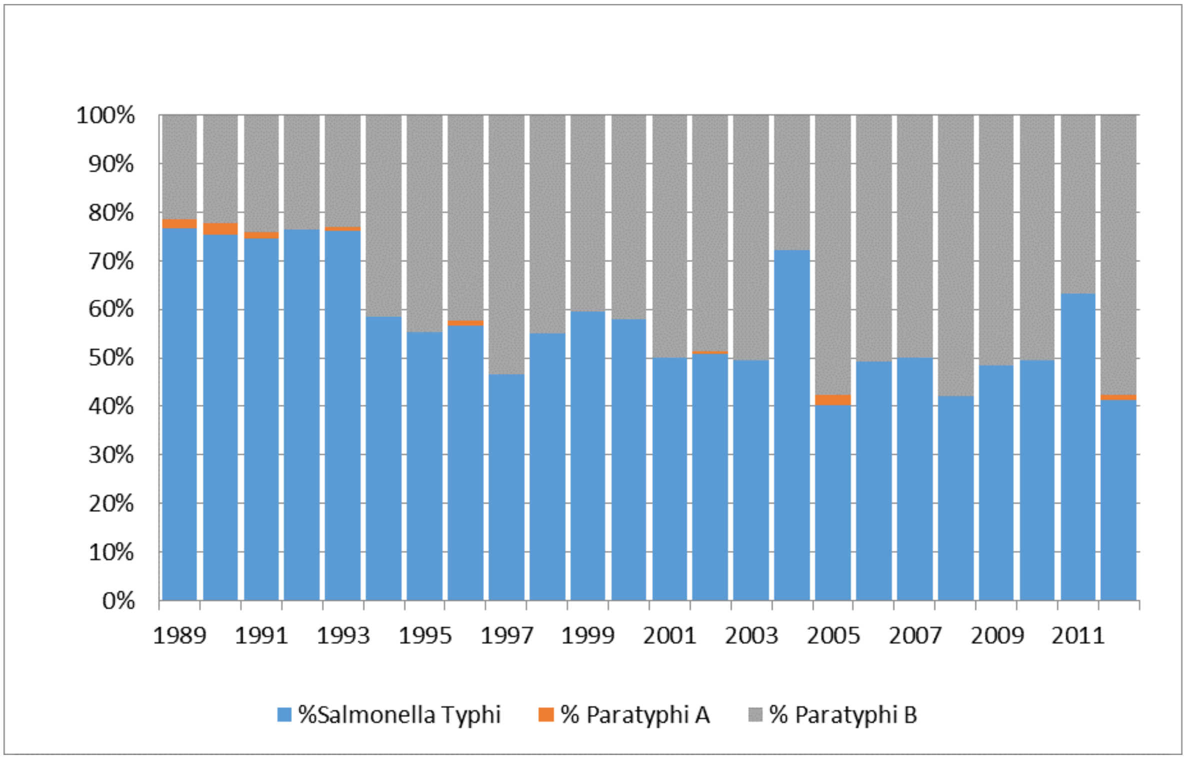

Supplementary Figure 5 Relative weight of Salmonella enterica serovar Tyhpi, Paratyphi A and Paratyphi B among TF cases in the Metropolitan Region 1989-2012

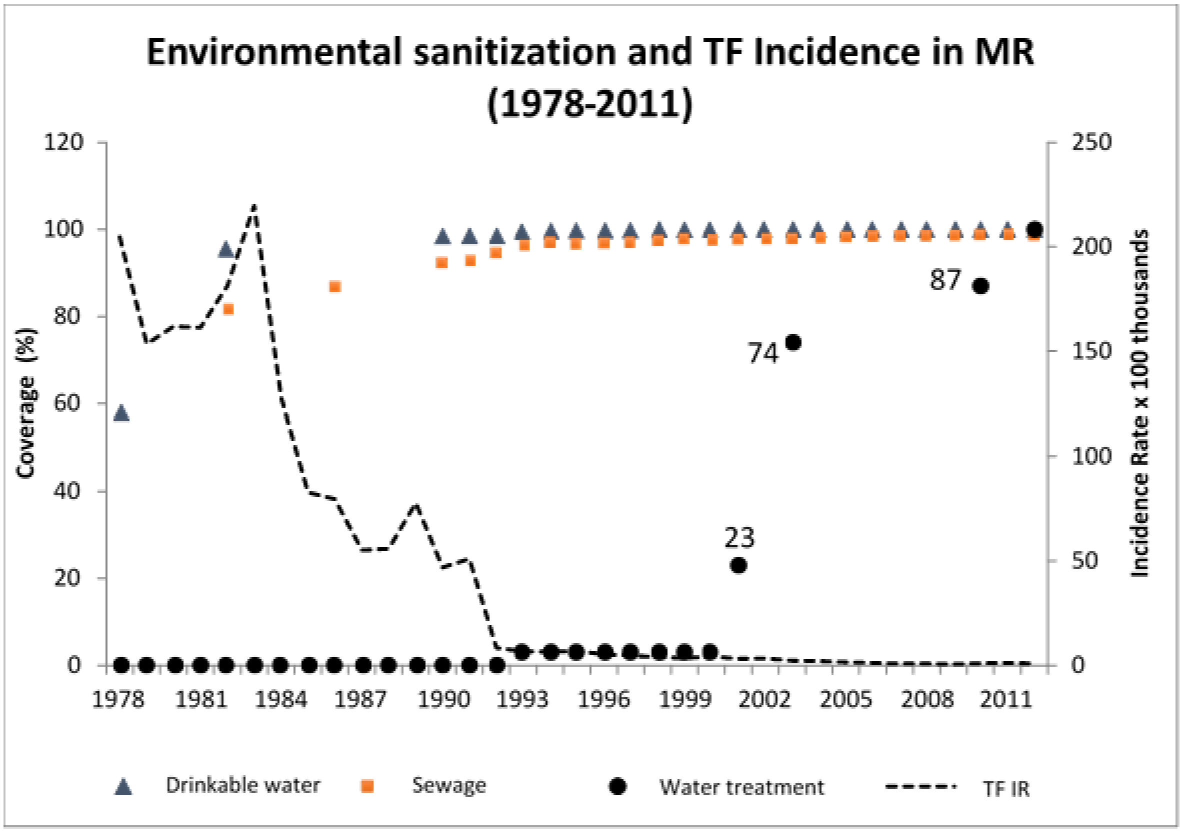

Supplementary Figure 6 Environmental Sanitation and Typhoid Fever incidence in the MR 1978-2012

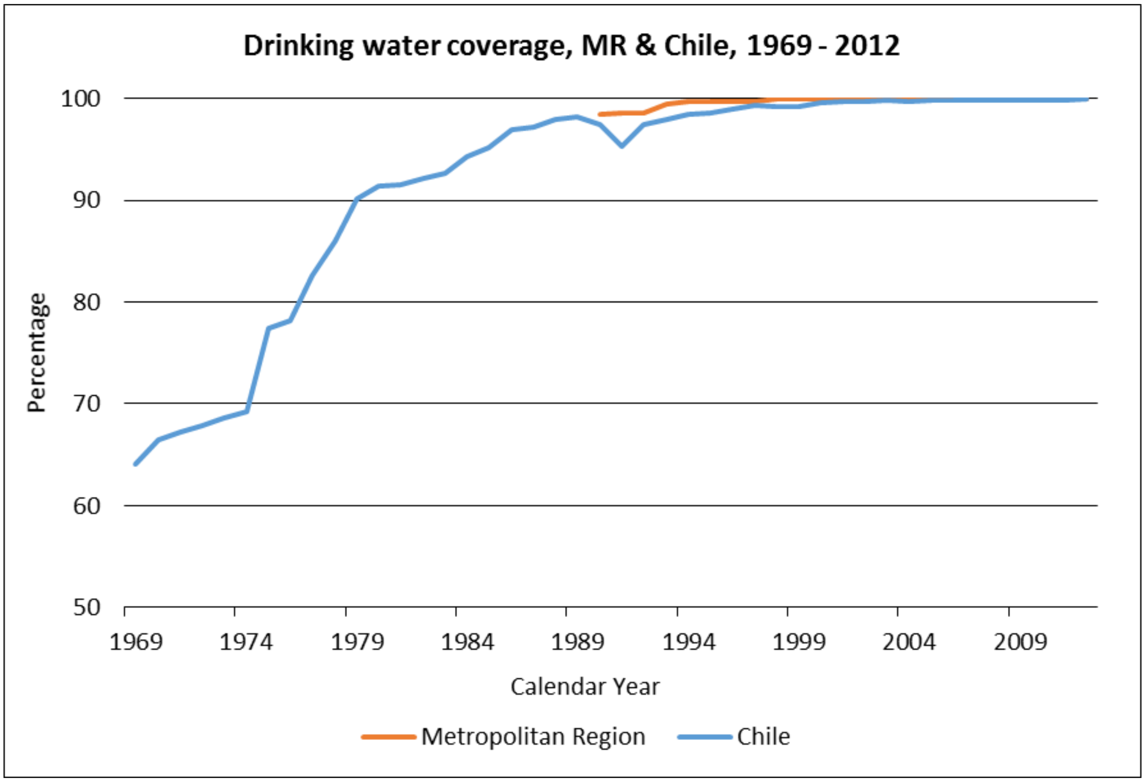

Supplementary Figure 7. Drinking water coverage in the Metropolitan Region and all of Chile 1969-2012.

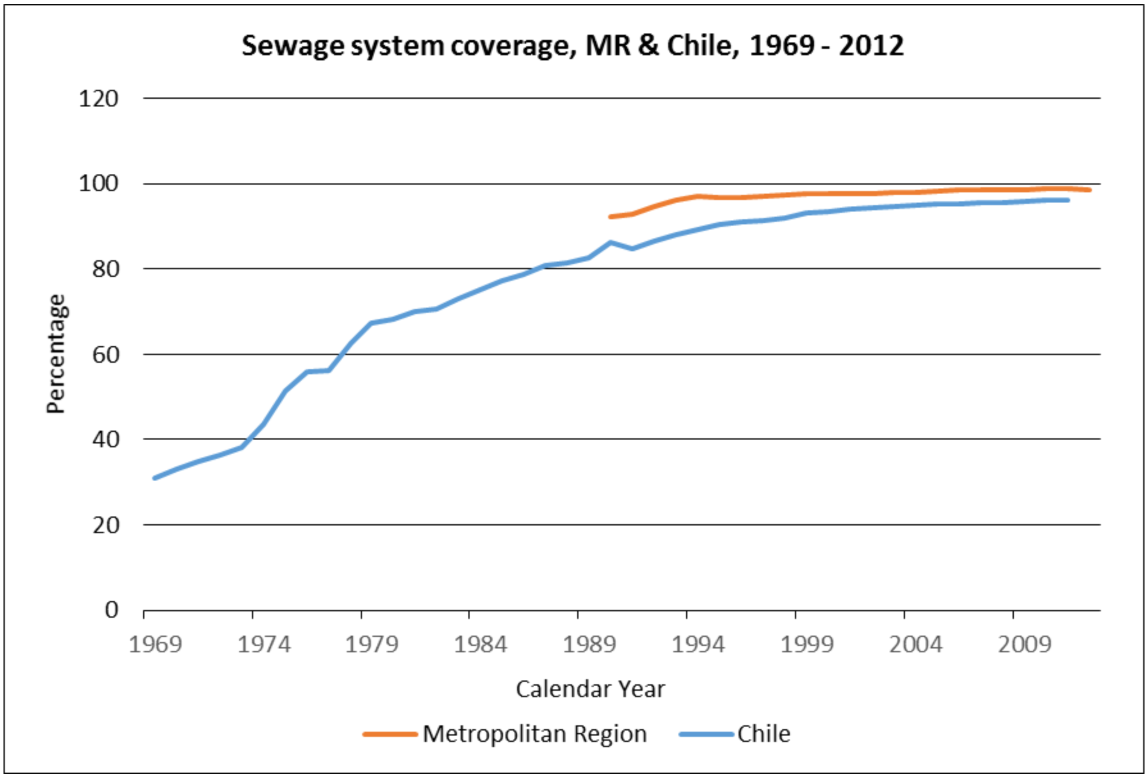

Supplementary Figure 8. Sewage system coverage in the Metropolitan Region and all of Chile 1969-2012.

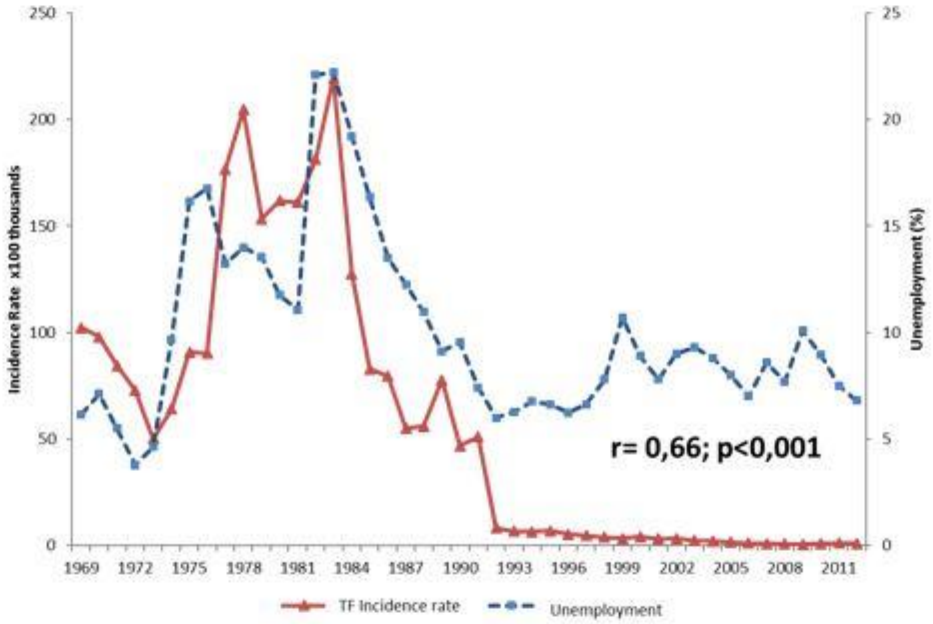

Supplementary Figure 9. Unemployment and Typhoid Fever Incidence in the Metropolitan Region of Chile 1969-2012.

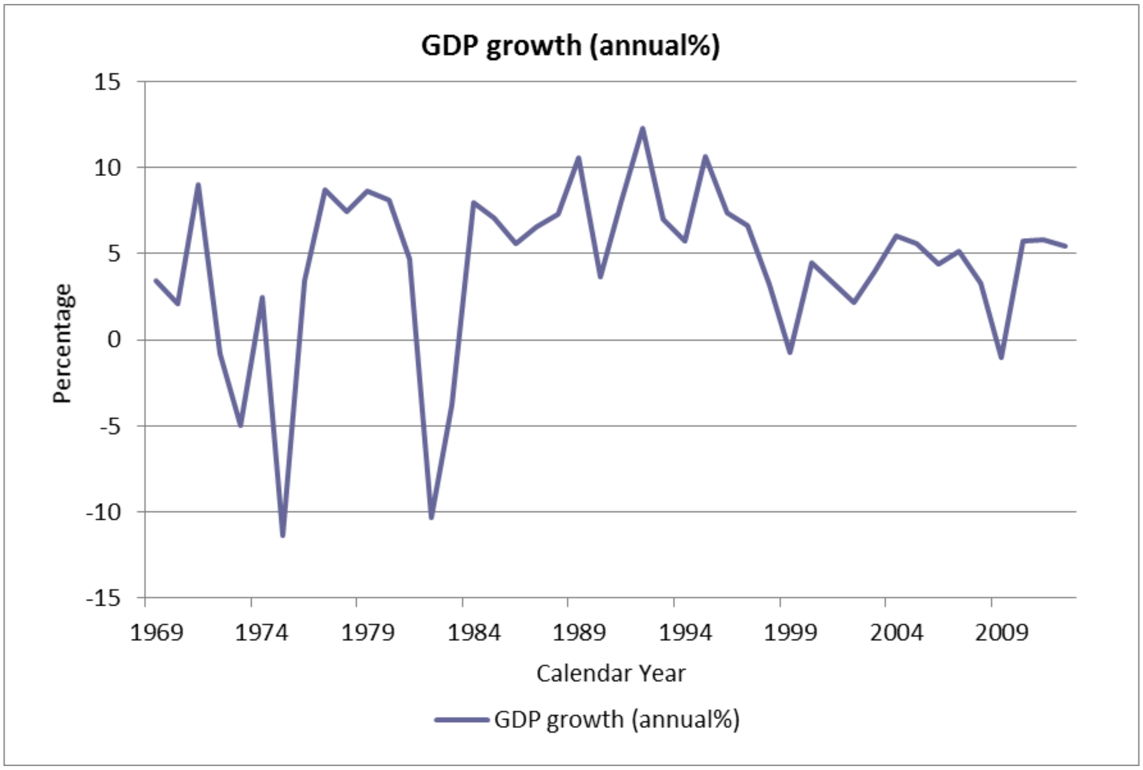

Supplementary Figure 10. GDP in the Metropolitan Region of Chile 1969-2012

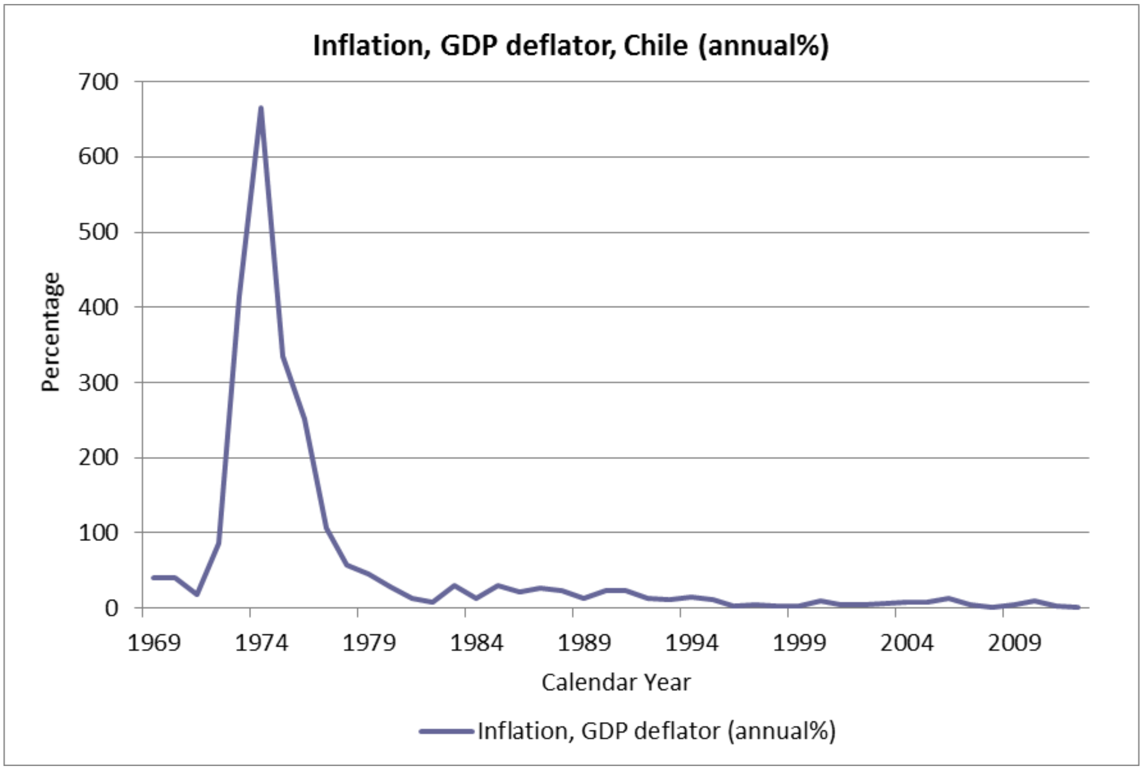

Supplementary Figure 11. Inflation, GDP deflator in Chile 1969-2012

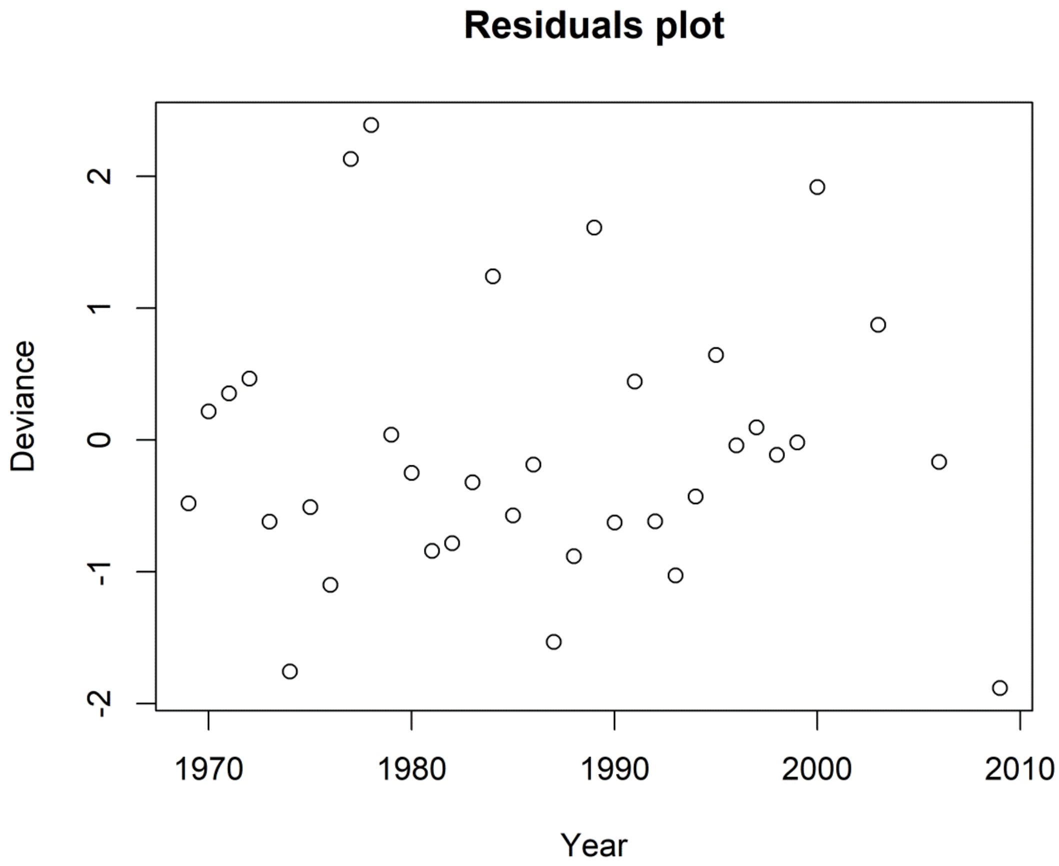

Supplementary Figure 12. Residuals of the selected ITS model for Typhoid Fever interventions. Metropolitan Region of Chile 1969-1972

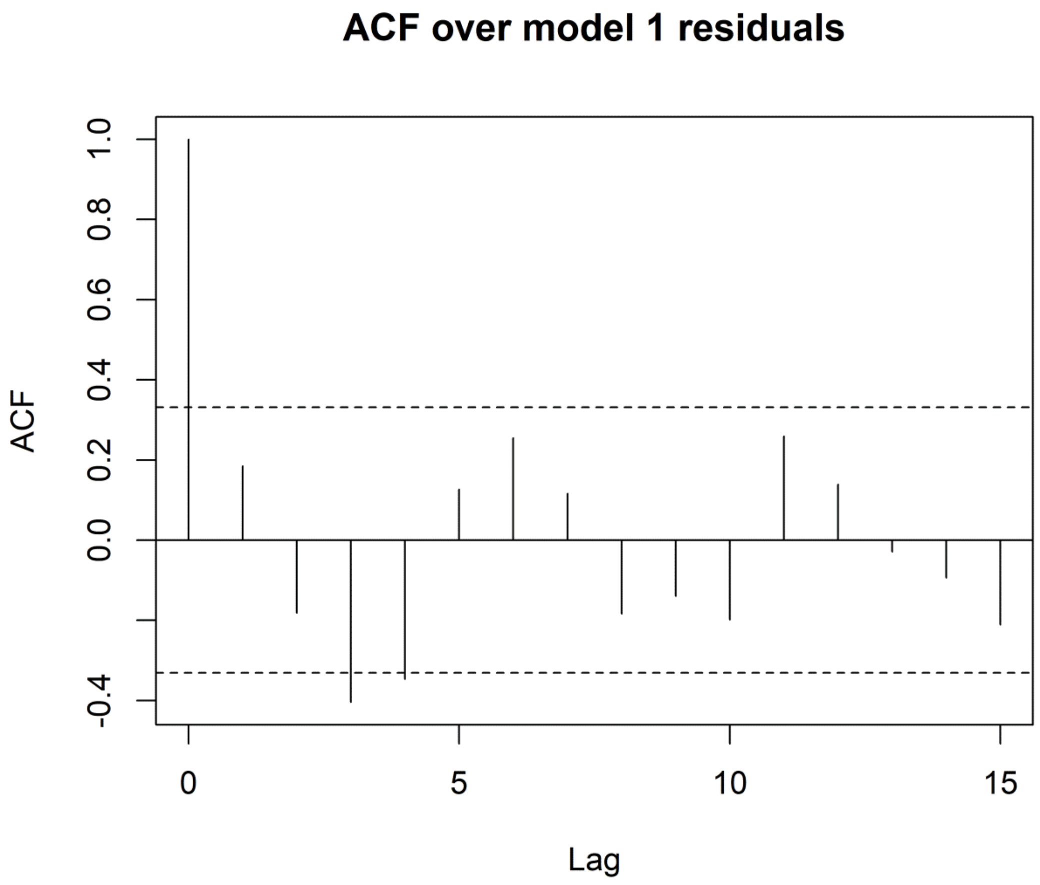

Supplementary Figure 13. Autocorrelation function (ACF) of residuals of the selected model for Typhoid Fever trend and effect of intervention

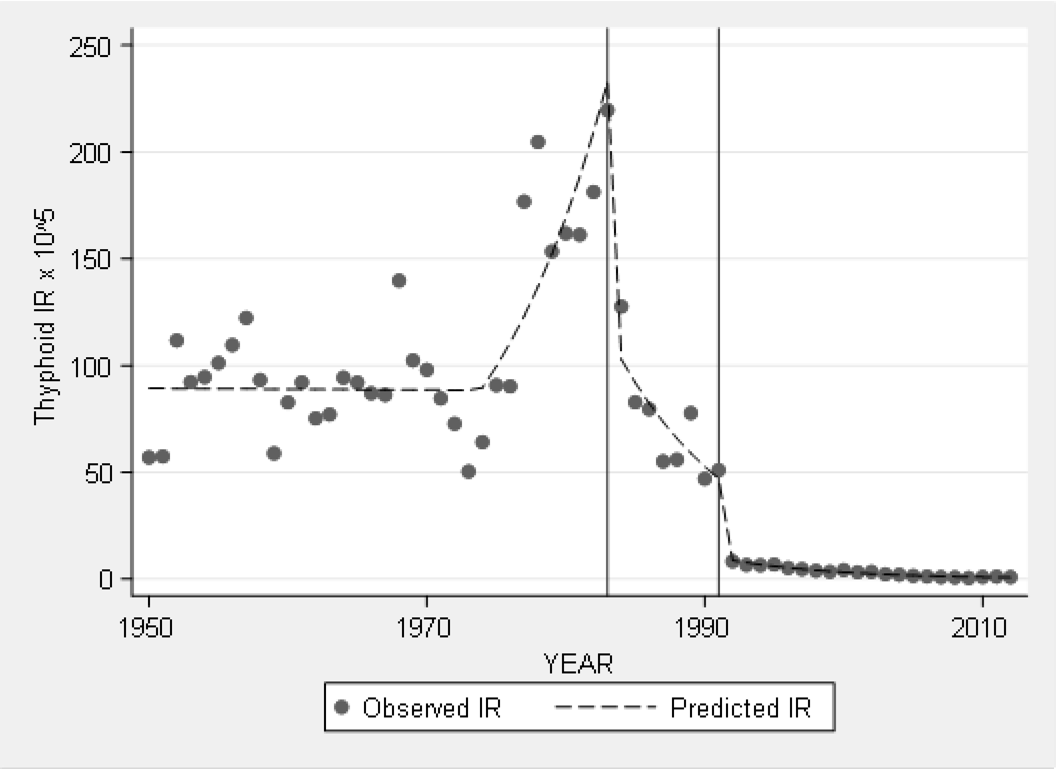

Supplementary Figure 14. ITS Model since 1950; this is new, sent today by email

SUPPLEMENTARY TABLE 2:
FITTED PARAMETERS WITH THE ITS MODELS USING 1950-2012 AND 1969-2012 TYPHOID FEVER INCIDENCE RATE DATA. MR

	Predicted Rates, per 100,000		Predicted Cases, number	
Year	ITS 1950-2012	ITS 1969-2012	ITS 1950-2012	ITS 1969-2012
1950	89,3		1567	
1951	89,2		1625	
1952	89,2		1683	
1953	89,2		1741	
1954	89,1		1799	
1955	89,1		1857	
1956	89,1		1932	
1957	89,0		2007	
1958	89,0		2082	
1959	89,0		2157	
1960	88,9		2233	
1961	88,9		2317	
1962	88,9		2402	
1963	88,8		2486	
1964	88,8		2571	
1965	88,8		2655	

1966	88,7		2740	
1967	88,7		2826	
1968	88,7		2911	
1969	88,6	111,8	2996	3780
1970	88,6	94,2	3081	3278
1971	88,5	79,4	3147	2823
1972	88,5	66,9	3212	2430
1973	88,5	56,4	3278	2090
1974	89,6	89,6	3387	3387
1975	99,6	99,6	3841	3841
1976	110,8	110,8	4351	4351
1977	123,2	123,2	4927	4927
1978	137,0	137,0	5577	5577
1979	152,3	152,3	6312	6312
1980	169,4	169,4	7141	7140
1981	188,3	188,3	8101	8101
1982	209,4	209,4	9188	9187
1983	232,9	232,8	10416	10415
1984	102,8	102,8	4689	4689
1985	92,0	92,0	4273	4273
1986	82,3	82,3	3909	3909
1987	73,6	73,6	3574	3574
1988	65,9	65,9	3266	3266
1989	58,9	58,9	2984	2984
1990	52,7	52,7	2724	2724
1991	47,1	47,1	2485	2485
1992	8,7	8,7	469	470
1993	7,7	7,7	422	422
1994	6,8	6,8	380	380
1995	6,0	6,0	341	341
1996	5,3	5,3	306	306
1997	4,7	4,7	275	275
1998	4,1	4,1	246	246
1999	3,7	3,7	221	221
2000	3,2	3,2	198	198
2001	2,8	2,8	177	177
2002	2,5	2,5	158	158
2003	2,2	2,2	141	141
2004	2,0	2,0	126	126
2005	1,7	1,7	112	112
2006	1,5	1,5	100	100
2007	1,3	1,3	89	89
2008	1,2	1,2	80	80
2009	1,0	1,0	71	71
2010	0,9	0,9	64	64

2011	0,8	0,8	57	57
2012	0,7	0,7	51	51
